# A Valuable Bill of Fare

**Published:** 1879-08

**Authors:** 


The best bill of fare for diabetics, which we have seen, has recently been given to the world by Mr. C. C. Waite, proprietor of the "Brevoort" and "Windsor" Hotels, in this city. It differs in some particulars from all lists of foods for this class of sufferers, to be found in the books, and its admitted value rests in the fact that each and every article herein recognized as allowable, has been tested and found useful by the author, and every article disallowed has been proved harmful by the same excellent authority. These facts render this schedule of foods very valuable to all diabetic patients, and impel us to present it to our readers in this issue of the JOURNAL OF HEALTH.

Mr. Waite decides, as we did long ago, that the disease alluded to is not to be cured by medicines. His experience clearly shows the uselessness of drugs, and the value of appropriate diet and regimen. He was reduced from strength to weakness, from vigorous health to death's door by this terrible disease before he knew its character. Then he sought counsel of the doctors and swallowed what they prescribed. He took steamer for Panama, attended by a physician, and returned lacking sufficient strength to walk from the carriage to his rooms in one of his hotels. He learned that Prof. Bouchardat, of Paris, was deemed the best authority in the world on the disease in question, and determined to sail for that city. The great French doctor examined him carefully, and told him that his salvation depended entirely upon himself; that medicines could avail little; that food, exercise, bathing, etc., were competent to save or slay him, according as they were wisely or unwisely used. He told him what to do, and how to live, and sent him back to America to recover, as it proved, and to assist many others in their efforts to rise from death to life.

Physicians will notice that Mr. Waite's bill of fare omits several articles which the majority of food-chemists deem advisable for diabetics. Dr. Donkin has written two books to show that skim-milk is a valuable agent in the treatment of diabetes. Why *skim-milk* instead of whole milk we could never understand. Pretty much all the lactose, or milk sugar, found in the milk, exists in the skimmed fluid and not in the cream. Mr. Waite commends cream, very properly, we think, and condemns milk. Nearly all writers allow tomatoes and celery; Mr. Waite permits the use of neither. All the doctors favor the use of tea; Mr. Waite declares it to be little less than poison to the diabetic. When asked why he opposes these articles, he tells us that they proved injurious to him. His plan has been to test all things, hold fast to that which has proved good, and put a black mark against anything which has proved harmful.

His hardest task was the effort to find a bread-stuff which at once met the demands of appetite and thoroughly agreed with him. Bouchardet told him to use gluten bread only, as being free from starch—starch being known to nourish the disease and not the patient. So he brought from Paris a quantity of Connor's gluten, and the bread made from it. He tried to live on it, but it was as dry as a chip, and tasted like medicine. All the delicious butter of the "Brevoort" and "Windsor" would not make it palatable. By accident he learned that a better gluten flour was made in New York. A guest of the "Brevoort"—the Hon. Frederick Billings—was told by Prof. Austin Flint that the gluten flour made by the HEALTH FOOD COMPANY, of 74 Fourth avenue, New York, was a very valuable food, and more acceptable to the palate than any of the foreign glutens. This fact was at once communicated to Mr. Waite, who lost no time in securing a quantity. The flavor pleased him, and its continued use proved very advantageous. So he places it at the head of the list of allowable foods, and uses it freely at each and every meal.

So firmly had the disease fastened itself upon him, so improbable did it seem that he should ever recover, so large was the circle of his acquaintance, and so general were the facts known, that his complete recovery excited great interest. It would have been scarcely less a miracle had one risen from the dead. Multitudes called upon him, and he was besieged with communications from the sick and suffering, asking the name of the wonderful medicine which had made him whole. All he could say was that medicine had very little to do with the cure. He freely told them, besides, that relief, perhaps cure, rested with themselves. He wrote out a good many food-lists, and added useful hints about Turkish baths, exercise, etc. The New York *Daily World*, which devotes a good deal of space to an intelligent discussion of food topics, got hold of one of these and printed it. It was republished in the medical journals, and has been adopted by many physicians, who look upon it as more trustworthy than any similar schedule to be found in their books.

In presenting this Bill of Fare to the readers of HALL'S JOURNAL OF HEALTH, and in expressing the decided opinion that it is in all respects superior to any food-list for diabetic sufferers which we have ever seen, and that the advice, so modestly conveyed, as to baths, exercise, etc., will prove of great value to patients of this class, we wish to call their attention to the vast good which intelligent laymen can accomplish by keeping a careful record of the facts connected with every case of prolonged suffering from wasting disease, and final recovery, and making it public.

